# Identifying Freshness of Spinach Leaves Stored at Different Temperatures Using Hyperspectral Imaging

**DOI:** 10.3390/foods8090356

**Published:** 2019-08-21

**Authors:** Susu Zhu, Lei Feng, Chu Zhang, Yidan Bao, Yong He

**Affiliations:** 1 College of Biosystems Engineering and Food Science, Zhejiang University, Hangzhou 310058, China; 2 Key Laboratory of Spectroscopy Sensing, Ministry of Agriculture and Rural Affairs, Hangzhou 310058, China

**Keywords:** hyperspectral imaging, spinach, freshness detection, visible/near-infrared spectra, near-infrared spectra

## Abstract

Spinach is prone to spoilage in the course of preservation. Spinach leaves stored at different temperatures for different durations will have varying degrees of freshness. In order to monitor the freshness of spinach leaves during storage, a rapid and non-destructive method—hyperspectral imaging technology—was applied in this study. Visible near-infrared reflectance (Vis-NIR) (380–1030 nm) and near-infrared reflectance (NIR) (874–1734 nm) hyperspectral imaging systems were used. Spinach leaves preserved at different temperatures with different durations (0, 3, 6, 9 days at 4 °C and 0, 1, 2 days at 20 °C) were studied. Principal component analysis (PCA) was adopted as a qualitative analysis method. The second-order derivative spectra were utilized to select effective wavelengths. Partial least squares discriminant analysis (PLS-DA), support vector machine (SVM), and extreme learning machine (ELM) were used to build models based on full spectra and effective wavelengths. All three models achieved good results, with accuracies above 92% for both Vis-NIR spectra and NIR spectra. ELM obtained the best results, with all accuracies reaching 100%. The overall results indicate the possibility of the freshness identification of spinach preserved at different temperatures for different durations using two kinds of hyperspectral imaging systems.

## 1. Introduction

Spinach (*Spinacia oleracea* L.) is a common vegetable which is abundant in carotenoids, vitamin C, vitamin K, minerals (calcium, iron, etc.), coenzyme Q10, and other nutrients [1]. In addition to its value as a food, spinach is also of rich medical value [2,3]. As a vegetable, spinach can be placed on the shelf after a simple processing following the harvest. However, spinach leaves have a large area and soft tissue, which requires high moisture, and the plant’s exuberant metabolism makes it easily lose freshness due to water loss in the course of picking, transportation, and sales. Furthermore, improper preservation can also cause deterioration of quality and greatly reduce the nutritional quality of spinach.

The freshness detection of spinach is important to ensuring its quality and commercial value. Generally, spinach leaves sold in markets are placed in both refrigerators and common shelves. The decay rates of spinach leaves under different temperatures are different, so spinach leaves can be stored for a longer time under lower temperature. A series of treatments for the freshness detection of samples are required in traditional detection methods, such as the measurement of chemical indexes like moisture, chlorophyll, and soluble sugar content [4,5]. However, traditional chemical detection methods are too cumbersome and require professional operators with expertise. The high cost of chemical tests also makes this approach not universally popular. Therefore, a rapid and non-destructive a method for the detection of vegetable freshness would be of great significance.

At present, machine vision technology and spectroscopy technology are widely used in agricultural product detection due to their non-destructive, rapid, and reliable characteristics. Machine vision technology can grade samples by analyzing the spatial information of samples and extracting relevant characteristics of agricultural product quality [6,7,8,9]. Machine vision technology can effectively detect the external quality of agricultural products (e.g., shape, color, etc.). However, spectroscopy technology is needed when it comes to the internal quality of agricultural products (e.g., protein content, sugar degree, acidity, etc.)—especially visible/near-infrared (Vis-NIR) spectroscopy [10,11]. The wavebands in visible/near-infrared spectroscopy reveal the spectral attributes of pigments, while wavebands in the near-infrared range are connected with the physical and chemical information of samples [12,13]. However, visible/near-infrared spectroscopy can only detect a small area at a time, which easily introduces data fluctuations and therefore inaccurate discriminant analyses.

Hyperspectral imaging technology can simultaneously acquire the spectral and spatial information of a research object, combining machine vision and spectroscopy technologies. Hyperspectral technology can simultaneously analyze the internal and external quality information of the sample [14,15]. Therefore, the application of hyperspectral imaging technology in the non-destructive detection of internal and external quality of fruits and vegetables has achieved good results in recent years. Visible/near-infrared spectra and near-infrared spectra can both be applied in the freshness detection. Elmasry et al. used a hyperspectral image system to detect apple bruises on different background colors, and partial least squares and stepwise discrimination analysis were used for the data analysis. The results indicated the possibility of apple bruise determination using the hyperspectral image system combined with chemometric methods [16]. Huang et al. applied hyperspectral transmittance images to discriminate insect-damaged soybeans. A 100% calibration accuracy was achieved by the support vector data description (SVDD) classifier [17]. Freshness monitoring using hyperspectral imaging technology also achieved decent results with both Vis-NIR and NIR spectra [18,19,20]. Although their study monitored spinach shelf-life with hyperspectral imaging (visible range, 400–1000 nm) through different packaging films, Lara et al. mainly focused on cold temperatures using only visible near-infrared spectra [19].

The objective of this study was to explore the feasibility of using hyperspectral imaging to detect the freshness of spinach leaves stored at different temperatures. The specific objectives were: (1) Explore the differences of spinach freshness detection using visible/near-infrared spectra and near-infrared spectra; (2) Verify the possibility of freshness detection under different temperatures (4 °C and 20 °C) preserved for different durations; (3) Compare the modelling performance of partial least squares discriminant analysis (PLS-DA), support vector machine (SVM), and extreme learning machine (ELM) models.

## 2. Materials and Methods

### 2.1. Sample Preparation

**Figure 1:**
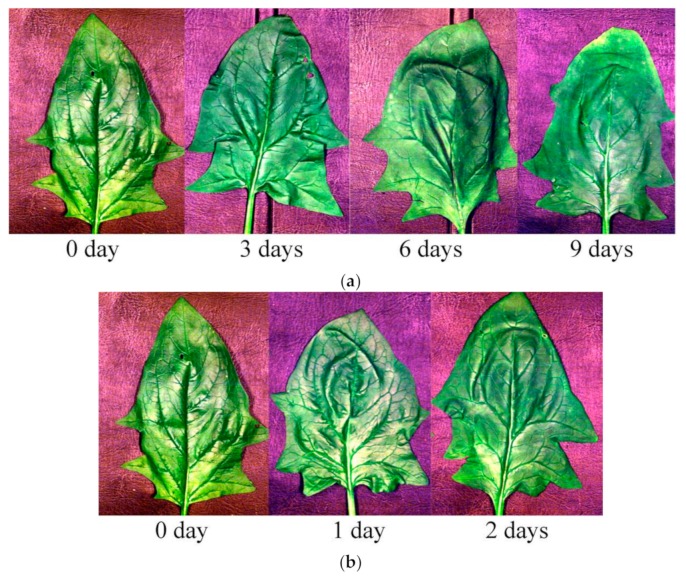
RGB images of spinach leaves preserved under different temperature conditions: (**a**) 4 °C; (**b**) 20 °C.

Fresh spinach leaves were directly harvested from a local farmland in Hangzhou, Zhejiang province, China. The spinach leaves were taken to the laboratory and cleaned for hyperspectral image acquisition. To evaluate the freshness of spinach, two different temperatures were selected for storage: 4 and 20 °C. For 4 °C storage, spinach leaves were stored in a refrigerator and covered with plastic film. The deterioration rates of spinach leaves under different temperatures are different. It is obvious that spinach leaves at 20 °C deteriorate much faster and cannot last as long as spinach leaves at 4 °C. Therefore, the storage duration for the two temperatures cannot be the same. Furthermore, the objective of this study was to verify whether the change of freshness under different temperatures could be detected, and the freshness of spinach leaves at 4 °C showed small differences in 3 days. Given this background, four storage durations were studied for spinach stored at 4 °C, including 0 days (which meant that the spinach samples were directly used for hyperspectral image acquisition after harvesting), 3 days, 6 days, and 9 days. For the 20 °C storage condition, spinach leaves were stored covered with plastic film. The set temperature (20 °C) was maintained by the air conditioner. Three time periods of storage were studied for spinach stored at 20 °C, including 0, 1, and 2 days. Figure 1 shows RGB images of spinach leaves stored at different temperatures for different durations. For each storage time duration, 30 spinach leaves were studied. In total, there were 120 and 90 spinach leaves prepared for 4 °C and 20 °C conditions, respectively. The samples of 0-days storage for the two storage temperatures were the same. The spinach leaves were divided randomly into a calibration set and a prediction set at the ratio of 2:1.

### 2.2. Hyperspectral Imaging System

Two hyperspectral imaging systems, including a near-infrared (NIR) hyperspectral imaging system covering the spectral range of 874–1734 nm and a visible/near-infrared (Vis-NIR) hyperspectral imaging system covering the spectral range of 380–1030 nm, were integrated in the same platform.

The NIR hyperspectral imaging system was composed of an imaging spectrograph (ImSpector N17E; Spectral Imaging Ltd., Oulu, Finland), a high-performance camera (Xeva 992; Xenics Infrared Solutions, Leuven, Belgium), and a camera lens (OLES22; Specim, Spectral Imaging Ltd., Oulu, Finland). The data cube of the NIR hyperspectral imaging system was 326 × λ × 256 (image width × image length × wavebands).

The Vis-NIR hyperspectral system comprised an imaging spectrograph (ImSpector V10E; Spectral Imaging Ltd., Oulu, Finland) with the spectral resolution of 2.8 nm, a high-performance CCD camera (Hamamatsu, Hamamatsu City, Japan), and a camera lens (OLES23; Specim, Spectral Imaging Ltd., Oulu, Finland). The data cube of the Vis-NIR hyperspectral imaging system was 672 × λ × 512 (image width × image length × wavebands).

For both systems, halogen light sources (Fiber-Lite DC950 Illuminator; Dolan Jenner Industries Inc., Boxborough, MA, USA) were used for illumination. A motion platform driven by an IRCP0076 electric displacement table (Isuzu Optics Corp., Taiwan, China) was used to move the samples for line-scanning. The acquisition of hyperspectral images was controlled by the corresponding software (Spectral Image-V10E and SpectralImage-Xenics 17E, Isuzu Optics Corp., Taiwan, China).

### 2.3. Hyperspectral Image Acquisition and Correction

In order to isolate samples from background easily, a black plate with low reflectance was used in this study. During the image acquisition, three parameters, including moving speed of conveyer belt, exposure time, and the height between the lens of the camera and the motion platform, were set first to prevent distortion of the hyperspectral images. For the NIR hyperspectral system, the three parameters were set as 25 mm/s, 5 ms, and 36 cm, respectively. For the Vis-NIR hyperspectral imaging system, the same parameters were set as 4.5 mm/s, 75 ms, and 36 cm, respectively.

The illumination source and the sensitivity of detector influence the accuracy of reflectance. Thus, the raw image (*I_raw_*) was calibrated by two reference standards according to the following equation:

(1) $$I_{c}=\;\frac{I_{raw}-I_{dark}}{I_{white}-I_{dark}}$$

where *I_c_* is the calibrated image; *I_dark_* represents the black reference image obtained by covering the lens with a lens cap whose reflectivity was about 0%; *I_white_* stands for the white reference image, obtained using a pure white Teflon board with a high reflectivity (nearly 100%).

### 2.4. Spectral Data Preprocessing and Extraction

For Vis-NIR hyperspectral images, pixel-wise spectra within spinach leaves contained obvious random noise in the head and end of the spectra. Thus, only the wavebands 101–460 (502–961 nm) were studied. Firstly, a binary image was obtained using a gray-scale image at 800 nm, in which the difference between the background reflectance and the sample reflectance was large. In the binary image, the leaf region was “1” and the background region was “0”. Then, the binary image was applied to the gray-scale image at each wavelength. The pixel-wise spectra within the leaf region were preprocessed by wavelet transform to reduce the random noise (wavelet function Daubechies 5 with decomposition level 3). An area normalization was applied to the preprocessed pixel-wise spectra to reduce the influence of the uneven surface of spinach leaves. The preprocessed pixel-wise spectra within one leaf were then averaged to represent the sample.

For NIR hyperspectral images, pixel-wise spectra in the spectral range of 975–1646 nm (200 wavebands) were studied. A binary image obtained using a gray-scale image at 1200 nm was applied to remove the background information by the same procedure mentioned above. The pixel-wise spectra within the leaf region were preprocessed by wavelet transform to reduce the random noise (wavelet function Daubechies 6 with decomposition level 3). An area normalization was also applied to the preprocessed pixel-wise spectra to reduce the influence of the uneven surface of spinach leaves. The average spectra were calculated to represent the samples.

### 2.5. Data Analysis Methods

#### 2.5.1. Principal Component Analysis

Principal component analysis (PCA) is a commonly used statistical method. A set of variables that may be related to each other can be transformed into a set of uncorrelated linear variables by the orthogonal transformation of PCA [21,22]. The converted variables are called the principal components (PCs). The first few PCs contain the majority of information of the hyperspectral image. PCA score scatter plots were formed to conduct the qualitative analysis of spinach leaves in this study.

#### 2.5.2. Effective Wavelength Selection

Raw hyperspectral images usually contain a great deal of uninformative information, which increases the volume of spectral data and influences the computation speed of models. To simplify the input of models and highlight the useful spectral information, the second-order derivative was used to select effective wavelengths in this study. The second-order derivative could weaken the background information of the spectra. The large differences in peaks and valleys observed in the second-derivative spectra revealed the variation of physical and chemical attributes among samples, which could be selected as effective wavelengths [23,24].

#### 2.5.3. Discriminant Models

Three discriminant methods, including partial least squares discriminant analysis (PLS-DA), support vector machine (SVM), and extreme learning machine (ELM), were applied to detect the freshness of spinach leaves.

PLS-DA is a multivariate statistical analysis method which can be used for discriminant analysis. PLS-DA can reduce the effects of multicollinearity between variables and reveal the linear relationship between the independent matrix (X) and the dependent variables (Y). Y stands for the specific category in the discrimination. The parameter of PLS-DA, the optimal number of latent variables, is selected according to leave-one-out cross-validation [24]. The categories of the spinach stored at 4 °C for PLS-DA were assigned as 0001, 0010, 0100, and 1000 (corresponding to spinach preserved for 0, 3, 6, and 9 days, respectively). The categories of the spinach stored at 20 °C for PLS-DA were numbered as 001, 010, and 100 (corresponding to spinach preserved for 0, 1, and 2 days, respectively).

SVM is a supervised method which is widely used for classification. The hyperplane created by SVM can isolate different samples with maximal margins. Linear and nonlinear data can all be dealt with efficiently with good results based on SVM methods. Compared with other methods, SVM always obtains good performance on small training sets. Radial basis function (RBF) is a widely used kernel function for SVM. The regularization parameter *c* and kernel function parameter *g* should be determined for the SVM model. Grid-search was used for parameter optimization in this study, and the search range varied from 2^−8^ to 2^8^ for both c and g [25,26].

As a widely used feedforward neural network, ELM can choose the weights connecting inputs to hidden nodes randomly with no need for modification, which brings a fast computation speed. The number of neurons in the hidden layer is the main parameter and needs to be determined for the ELM model. There are various activation functions that can be applied in ELM models (e.g., sigmoid function, sine function, radial basis function, etc.). Radial basis function (RBF) was selected as the activation function in this study. The optimal number of neurons was chosen according to the performance of ELM models using different numbers of neurons [27,28]. The categories of the spinach stored at 4 °C for SVM and ELM were assigned as 1, 2, 3, and 4 (corresponding to spinach preserved for 0, 3, 6, and 9 days, respectively). The categories of the spinach stored at 20 °C for SVM and ELM were numbered as 1, 2, and 3 (corresponding to spinach preserved for 0, 1, and 2 days, respectively).

### 2.6. Software and Model Evaluation

Classification accuracy, which was defined as the ratio of the number of correctly classified samples to the total number of samples, was used to evaluate the performance of models. PLS-DA, SVM, and ELM models were all built based on Matlab R2014b (The Math Works, Natick, MA, USA).

## 3. Results

### 3.1. Spectral Profile

**Figure 2:**
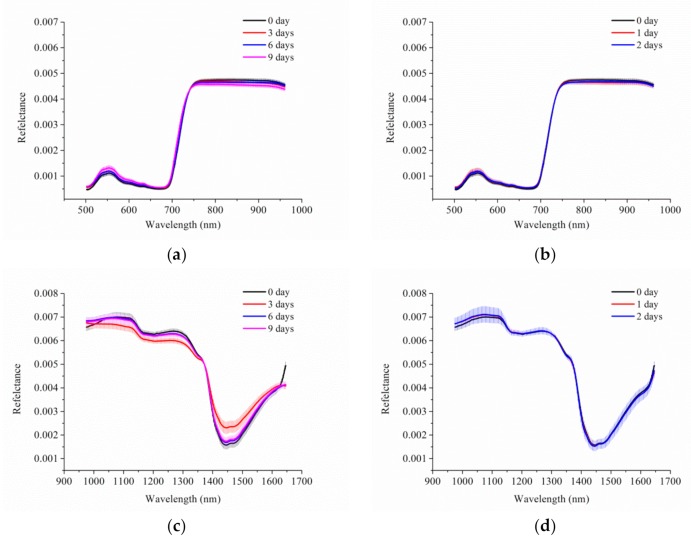
The average spectra with standard deviation (SD): visible/near-infrared (Vis-NIR) hyperspectral images of spinach leaves stored at (**a**) 4 °C and (**b**) 20 °C; NIR hyperspectral images of spinach leaves stored at (**c**) 4 °C and (**d**) 20 °C.

Figure 2 shows the average spectra with vertical bars, which represent standard deviation (SD) of Vis-NIR and NIR hyperspectra of spinach leaves used for calibration. For the Vis-NIR spectra, the change tendency of spinach leaves stored at different temperatures with different durations were all similar. The same curve change tendency was also observed in NIR spectra for spinach leaves preserved at 4 and 20 °C. The SD of the spectra of spinach leaves stored for different days was similar, and could not be distinguished easily.

There were also some overlaps of spectra among spinach leaves stored for different durations for both Vis-NIR spectra and NIR spectra. In order to obtain distinct information used for the freshness detection of spinach leaves, further processing should be applied to the spectral data.

### 3.2. PCA Scores Scatter Plot Analysis

**Figure 3:**
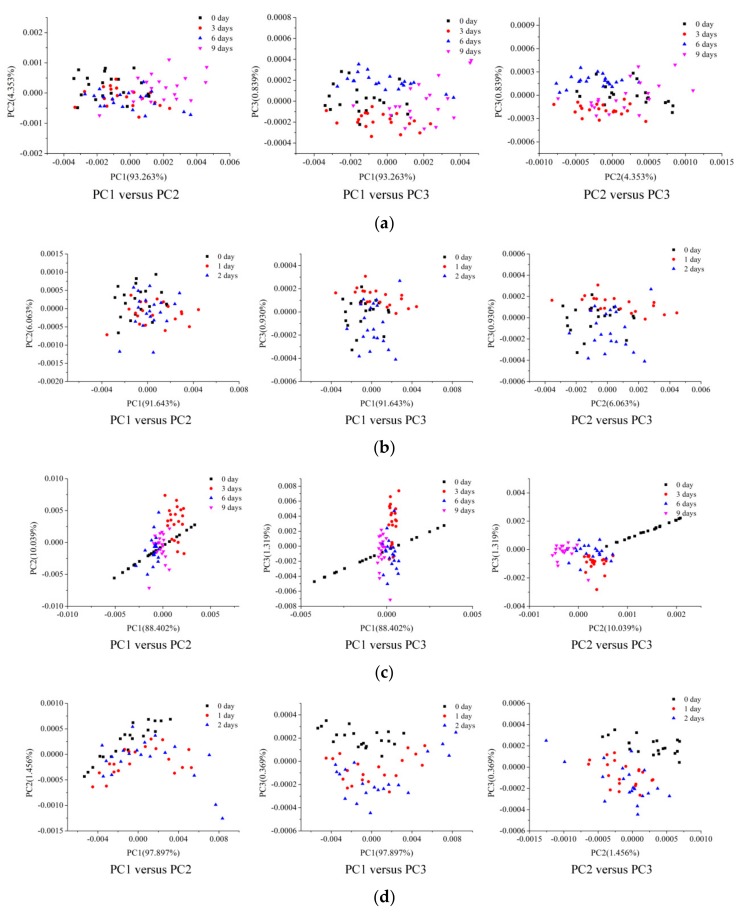
Score scatter plots of spinach leaves stored at (**a**) 4 °C and (**b**) 20 °C based on Vis-NIR hyperspectral data. Score scatter plots of spinach leaves stored at (**c**) 4 °C and (**d**) 20 °C based on NIR hyperspectral data.

PCA scores scatter plot analysis was applied to show intuitive differences in spinach leaves with different freshness levels. The first three PCs explained more than 98% of the variance in the spectral data of spinach leaves, and the first three PCs were thus used to form the scores scatter plot. Spinach leaves of the classification set were used to conduct the PCA. For the score scatter plot of spinach leaves stored at 4 °C (Figure 3a) and 20 °C (Figure 3b) based on Vis-NIR hyperspectral data, spinach leaves stored at different temperatures for different durations clustered together according to their own attributes, and some overlaps among different groups could be found. Compared with the scores scatter plot based on Vis-NIR spectra, the scores scatter plot of spinach stored at 4 °C based on NIR spectra (Figure 3c) showed more distinct attributes of different clusters, with fewer overlaps among the four categories. From Figure 3d, there were also some overlaps among the three groups, making it difficult to distinctly discriminate spinach stored at 20 °C with different durations based on NIR spectra.

In order to distinguish the freshness of spinach leaves more specifically, quantitative analyses combined with chemometric methods should be introduced.

### 3.3. Effective Wavelength Selection

**Figure 4:**
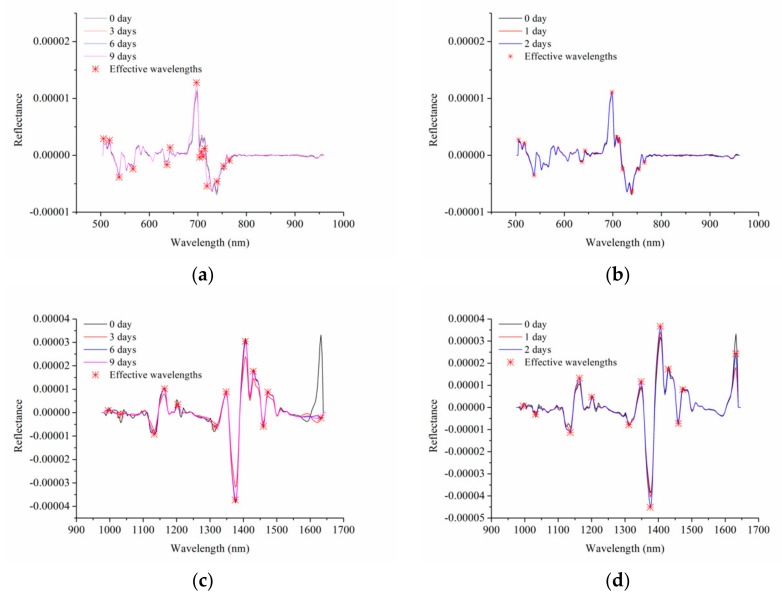
Effective wavelengths selected by second-order derivative spectra of spinach leaves stored at (**a**) 4 °C and (**b**) 20 °C based on Vis-NIR hyperspectral data. Effective wavelengths selected by second-order derivative spectra of spinach leaves stored at (**c**) 4 °C and (**d**) 20 °C based on NIR hyperspectral data.

Full spectra usually contain a great deal of useless information (e.g., redundancy, collinearity, and background information), which increases the computation time and affects the robustness of models. To decrease the data volume and improve the model building speed, the second-order derivative spectra of spinach leaves used for calibration were used for effective wavelengths selection. As shown in Figure 4, the peaks and valleys with lager differences among different groups were marked as effective wavelengths. Table 1 summarizes the selected effective wavelengths chosen for model building. A total of 15 and 12 effective wavelengths were selected for spinach stored at 4 °C and 20 °C using Vis-NIR hyperspectral imaging, respectively, and 14 and 13 effective wavelengths were selected for spinach stored at 4 °C and 20 °C by NIR spectra, respectively. More than 90% data volume was decreased by selecting effective wavelengths. Effective wavelengths are related to some chemical substances in the spinach. For spinach leaves stored under different temperatures, most of the effective wavelengths selected by Vis-NIR or NIR were the same, which implies the stability of the selection of effective wavelengths. With the change of freshness, the pigment content in the leaves will also change. Therefore, the effective wavelengths selected by Vis-NIR are in close relation to the leaf colors. For example, 538 nm and 566 nm wavelengths are linked with anthocyanin absorption, and the effective wavelength at 700 nm is related to chlorophyll content [29]. NIR spectra reveal more information about the change of chemical substances in the spinach. Wavelengths at 988 nm [30], 1032 nm [6], 1136 nm, and 1164 nm closely correspond to water content in the spinach leaves, while 995, 1311, 1132, 1321, 1348, 1406, 1473, and 1511 nm are in relation to organics [31,32]. For example, 995 nm represents the second vibration of N–H bonds in proteins or amino acids [33]. The wavelength of 1311 nm corresponds to the first overtone of the O–H stretch and O=C=O bending [34]; 1321 nm is related to O–H, C–H, and N–H bonds [35]; 1348 nm is attributed to the first overtone of amide B with the fundamental amide II and III vibrations, while 1473 nm is ascribed to OH, CH, and CH_2_ deformations [34]. It can be observed that most of the selected effective wavelengths are related to water, protein (amino acid), and fat. It is conceivable that the content of these substances in the spinach will change with the decrease of freshness, which is why these wavelengths were selected as effective wavelengths.

### 3.4. Classification Models Using Vis-NIR and NIR Spectra

Classification models using Vis-NIR and NIR spectra were established with full spectra and effective wavelengths, and results of the four circumstances are presented in Table 2 and Table 3. Generally speaking, although the model establishment using full spectra required more computation time, models based on full spectra performed better than those based on effective wavelengths, with all accuracies being over 97.5% and most of them reaching 100%. Satisfactory results were obtained under most circumstances, with accuracy surpassing 90%, indicating the feasibility of applying hyperspectral imaging in spinach freshness detection.

For models using the Vis-NIR spectra, it can be seen from Table 2 that all three models based on the full spectra could reach a completely accurate classification, with the exception of the SVM model on the calibration set in the 20 °C sample set. Nevertheless, the SVM model still achieved 98.33% classification accuracy for this calibration set. Models based on the effective wavelengths also obtained decent results, with most accuracies over 92.5%. ELM models again reached a 100% accurate classification in both 4 °C and 20 °C sample sets. The PLS-DA models performed second best next to the ELM models. Misclassification occurred in the prediction of the 20 °C sample set using effective wavelengths, while classification accuracies were all 100% in other cases. SVM models performed worst, without any accuracy reaching 100% for both calibration and prediction sets.

For models using the NIR spectra, it can be observed from Table 2 that similar results were obtained when full spectra were adopted. All three models based on the full spectra reached a completely accurate classification except for the SVM model on the prediction set in the 4 °C sample set, but its 97.5% accuracy was fairly acceptable. In contrast to models using the Vis-NIR spectra, all the models based on the effective wavelengths accomplished 100% classification accuracy in the 20 °C sample set. Regarding the 4 °C sample set, ELM models were still the best, with all accuracies reaching 100%. PLS-DA models were still second-best compared to ELM models, followed by the SVM models. It was also observed that models based on effective wavelengths achieved accuracies of at least 92.5%, while this number was 86.67% for the Vis-NIR spectra, indicating a better performance of NIR spectra than that of Vis-NIR in classification with effective wavelengths.

The ELM models obtained the most satisfactory results, with all accuracies reaching 100% under the four circumstances. PLS-DA models were inferior to the ELM models but better than the SVM models. Moreover, it could be deduced from the two tables that NIR spectra and Vis-NIR spectra could all be applied to monitor the freshness of spinach leaves, which was consistent with the good results achieved in some existing studies [18,19,20]. Giovenzana et al. monitored the freshness decay of fresh-cut *Valerianella locusta* L. during storage at different temperatures (4, 10, and 20 °C), and residual predictive deviation (RPD) values <3 were obtained for water content and pH [18]. Lara et al. also obtained good results in the course of monitoring spinach shelf-life with hyperspectral imaging in the Vis-NIR spectral range through packaging films stored at 4 °C for 21 days [19]. Apart from Vis-NIR spectra, NIR spectra also could obtain satisfactory results. Sánchez evaluated the ability of NIR spectra to discriminate green asparagus stored in refrigerated storage (2 °C) for different durations (7, 14, 21, and 28 days). Partial least squares 2-discriminant analysis (PLS2-DA) models were developed for classification, and the accuracies were between 81% and 100% [20].

In summary, the results in the tables prove the feasibility of the application of hyperspectral imaging in spinach freshness detection, and demonstrate that models based on effective wavelengths can also obtain good results.

## 4. Conclusions

Spinach leaves deteriorated at different speeds in different preservation situations. Vis-NIR and NIR spectra were applied to detect the freshness of spinach preserved at different temperatures (4 and 20 °C) for different durations (0, 3, 6, and 9 days for 4 °C and 0, 1, and 2 days for 20 °C). PCA was conducted on spectral reflectance and PCA scores scatter plots were also obtained for qualitative analyses in this study. From PCA scores scatter plots, differences of different categories could be observed but overlaps of scatters still existed, which required further analyses. The second-order derivative spectra were used for the selection of effective wavelengths. PLS-DA, SVM, and ELM models using full spectra and effective wavelengths were established. Decent results were obtained by all three models for spinach leaves stored at different temperatures and durations based on two kinds of hyperspectral systems. Among all three models, ELM models performed best, with all accuracies reaching 100% for the two spectral systems in the freshness detection of spinach leaves preserved at 4 °C and 20 °C. 

In sum, the overall results demonstrate that both Vis-NIR and NIR spectra could be used for the freshness monitoring of spinach at different temperatures for different storage times. Although the results indicated the feasibility of detecting the freshness of spinach leaves using both kinds of hyperspectral systems, the data processing procedures were laborious, which is an obstacle of practical application. Additionally, to further improve the robustness of the model, the sample number should be extended and different varieties of samples should also be taken into consideration in future studies.

## Figures and Tables

**Table 1:** Corresponding effective wavelengths selected by second-order derivative spectra.

Hyperspectral Imaging System	Temperature (°C)	No.	Effective Wavelengths (nm)
Vis-NIR	4	15	506, 518, 538, 566, 636, 643, 697, 704, 707, 711, 714, 719, 739, 753, 765
	20	12	506, 518, 538, 636, 643, 698, 709, 714, 720, 739, 753, 765
NIR	4	14	988, 1032, 1132, 1164, 1204, 1321, 1348, 1375, 1406, 1429, 1460, 1473, 1511, 1632
	20	13	995, 1032, 1136, 1164, 1200, 1311, 1348, 1375, 1406, 1429, 1460, 1473, 1632

**Table 2:** Results of classification models using Vis-NIR spectra based on full spectra and effective wavelengths.

Temperature (°C)	Classifier	Parameter ^1^	Full Spectra (%)		Parameter	Effective Wavelengths (%)	
			Calibration	Prediction		Calibration	Prediction
4	PLS-DA	12	100	100	11	100	100
	SVM	(108, 1)	100	100	(106, 102)	95	92.5
	ELM	10	100	100	11	100	100
20	PLS-DA	11	100	100	12	100	96.67
	SVM	(106, 102)	98.33	100	(104, 105)	95.00	86.67
	ELM	13	100	100	19	100	100

^1^ Parameter means the parameters of partial least squares discriminant analysis (PLS-DA), support vector machine (SVM), and extreme learning machine (ELM) models with optimal performances. The parameter for PLS-DA is the optimal number of latent variables; the parameters for SVM models are the regularization parameter *c* and kernel function parameter *g*; the parameter of the ELM model is the number of hidden layer neurons.

**Table 3:** Results of classification models using NIR spectra based on full spectra and effective wavelengths.

Temperature (°C)	Classifier	Parameter ^1^	Full Spectra (%)		Parameter	Effective Wavelengths (%)	
			Calibration	Prediction		Calibration	Prediction
4	PLS-DA	10	100	100	10	98.75	100
	SVM	(106, 103)	100	97.50	(106, 104)	98.75	92.50
	ELM	12	100	100	18	100	100
20	PLS-DA	4	100	100	4	100	100
	SVM	(103, 103)	100	100	(103, 105)	100	100
	ELM	7	100	100	8	100	100

^1^ Parameter means the parameters of partial least squares discriminant analysis (PLS-DA), support vector machine (SVM), and extreme learning machine (ELM) models with optimal performances. The parameter for PLS-DA is the optimal number of latent variables; the parameters for SVM models are the regularization parameter *c* and kernel function parameter *g*; the parameter of the ELM model is the number of hidden layer neurons.
